# Effects of a modular telehealth intervention on comorbid conditions in service members with mild traumatic brain injury

**DOI:** 10.3389/fneur.2025.1594748

**Published:** 2025-07-29

**Authors:** Jesse R. Fann, Tessa Hart, Kathleen R. Bell, Wesley Cole, Sonia Jain, Rema Raman, Jason Barber, Sureyya Dikmen, John Richardson, Murray B. Stein, Nancy Temkin

**Affiliations:** ^1^Department of Psychiatry and Behavioral Sciences, University of Washington, Seattle, WA, United States; ^2^Department of Rehabilitation Medicine, University of Washington, Seattle, WA, United States; ^3^Moss Rehabilitation Research Institute, Elkins Park, PA, United States; ^4^Department of Physical Medicine and Rehabilitation, University of Texas Southwestern, Dallas, TX, United States; ^5^Department of Brain Injury Medicine, Womack Army Medical Center, Fort Bragg, NC, United States; ^6^Herbert Wertheim School of Public Health and Human Longevity Science, University of California, San Diego, La Jolla, CA, United States; ^7^Alzheimer’s Therapeutic Research Institute, University of Southern California, Los Angeles, CA, United States; ^8^Department of Neurological Surgery, University of Washington, Seattle, WA, United States; ^9^Department of Health Management and Policy, University of Michigan, Ann Arbor, MI, United States; ^10^Department of Psychiatry, University of California, San Diego, La Jolla, CA, United States; ^11^VA San Diego Healthcare System, San Diego, CA, United States; ^12^Department of Biostatistics, University of Washington, Seattle, WA, United States

**Keywords:** traumatic brain injury, concussion, comorbidity, telehealth, clinical trial

## Abstract

**Objective:**

Many active duty service members with mild traumatic brain injury (mTBI) report comorbidities such as depression, anxiety, PTSD, insomnia, and pain. We analyzed data from a prior randomized controlled trial (RCT) to examine the effects of evidence-based treatment modules, delivered by telephone, on the number and symptom burden of five common comorbidities.

**Setting and participants:**

356 service members from two military medical centers who had sustained deployment-related mTBI in the preceding 2 years.

**Design:**

Secondary analysis of RCT comparing 6 months of telephone-delivered problem-solving treatment (PST) with comorbidity-specific modules to education only (EO).

**Main measures:**

Comorbidity burden measured by Patient Health Questionnaire-9, Brief Symptom Inventory-Anxiety, PTSD Checklist, Pittsburgh Sleep Quality Inventory, Rivermead Postconcussion Symptoms Questionnaire (headache item) assessed at baseline and 6 and 12 months.

**Results:**

47% of service members endorsed ≥ 3 comorbidities at baseline. At 6 months, the PST group had significantly fewer comorbidities, greater improvement in depression, anxiety, PTSD, and sleep, but not headache, and higher response/remission rates for depression and sleep, compared to EO. There were no significant group differences at 12 months.

**Conclusion:**

Telephone-delivered PST with comorbidity-specific modules reduces burden of comorbidities after deployment-related mTBI. Research is needed on how to maintain improvements over time.

## Introduction

Over more than 20 years of U.S. military engagement in Operations Enduring Freedom, Iraqi Freedom, and New Dawn (OEF/OIF/OND), many service members sustained at least one mild traumatic brain injury (mTBI), often leading to persisting neuropsychiatric difficulties. However, many questions remain about the best approaches to treating these challenges in Veterans, particularly those with deployment-related mTBI. Veterans with mTBI have a higher prevalence of comorbid conditions such as post-traumatic stress disorder (PTSD), depression, anxiety, and substance use disorders compared to those without TBI (1). Other common comorbidities include sleep disturbance (2) and pain (3), especially headache (4, 5). These problems often co-occur and may persist for months or years. In a population-based study of OEF/OIF/OND Veterans with any TBI, almost one-third had one psychiatric diagnosis and half had two or more diagnoses (6). In both military and civilian samples, TBI accompanied by these comorbid mental health problems is associated with impaired coping, functional impairment, and decreased quality of life (5, 7–9).

The treatment of mTBI and its mental and physical comorbidities was noted by the Institute of Medicine as a critical area of focus among service members and Veterans (10). However, a systematic review of treatment approaches for deployment-related psychiatric conditions that are comorbid with mTBI revealed insufficient evidence to support treatment guidelines, despite the fact that such guidelines exist for PTSD, depression, and other conditions in the absence of TBI (11). It was noted that some psychotherapies designed for the general population do result in symptom reduction in those with mTBI; however, many military personnel fail to follow through with treatment recommendations due to perceived stigma, lack of access to care, and other barriers (12–14). Thus, there remains a need for flexible, accessible treatments to address the comorbidities that accompany mTBI in this population (15).

In the randomized controlled CONTACT (Concussion Treatment after Combat Trauma) study, we compared the effects of a multifaceted problem-solving treatment (PST) delivered by telephone versus education only (EO) in a group of service members with deployment-related mTBI (16). The PST group showed significantly more improvement on a measure of psychological distress after 6 months of treatment, but reduction in postconcussive symptoms did not differ by group (17). Although the PST treatment arm included evidence-based treatment modules targeting several commonly occurring comorbidities, the effects on comorbidities was not directly examined.

This secondary analysis seeks to investigate whether the person-centered, modular components of the treatment intervention reduce comorbidity burden in this population. While the primary study focused on group differences in overall psychological distress and postconcussive symptoms, the current analysis focuses on the presence, severity, and response rates of five common individual comorbidities (depression, anxiety, PTSD, insomnia, and headache), thus providing a finer-grained view of the efficacy of a novel, phone-based intervention at the individual level. Specifically, we examined the effects of the person-centered intervention package that included a system of detection, education, and therapeutic support tailored to address identified symptoms among the five comorbid conditions. We hypothesized that, although participation in the CONTACT study did not require the presence of particular symptoms, (1) participants in both treatment arms would endorse a high burden of comorbid conditions at baseline; and (2) those enrolled in the PST intervention, with modules addressing specific comorbidities, would report significantly lower comorbid symptom burden and decreased symptom severity compared to those in the EO condition, both immediately after 6 months of treatment and at 12-month follow-up.

## Materials and methods

The study was approved by the participating academic and military institutional review boards and all participants gave informed consent. Comprehensive study procedures, including inclusion and exclusion criteria, descriptions of measures, and specifics regarding the intervention are detailed elsewhere (16). The clinical trial was registered at: clinicaltrials.gov, identifier NCT01387490.

### Participants

In brief, participants were 356 service members from the TBI Clinics of two military medical centers (Madigan Army Medical Center and Womack Army Medical Center) who had sustained deployment-related mTBI during OEF/OIF/OND within the previous 2 years. Participants were excluded for moderate to severe TBI requiring hospitalization, psychosis, active suicidal ideation, or participation in a formal TBI treatment program on base.

### Measures

The five comorbid conditions (depression, anxiety, PTSD, insomnia, and headache) were assessed at baseline, after 6 months of treatment, and at 12-month follow-up. Depression was assessed using the Patient Health Questionnaire-9 (PHQ-9); anxiety with the Brief Symptom Inventory (BSI) Anxiety subscale; PTSD with the PTSD Checklist–Military version (PCL-M); insomnia with the Pittsburgh Sleep Quality Index (PSQI); and headache with the headache item from the Rivermead Postconcussion Symptoms Questionnaire (RPQ). Table 1 also shows the measures (if different) that were used to track symptoms during intervention, the cutoff scores used to determine the presence of each comorbidity, and the cutoff scores when a specialty referral was recommended. Basic demographic information, TBI-related information, and additional clinical measures (for use as secondary outcome measures not pertinent to the current analysis) were also collected at baseline (16).

**Table 1:** Outcome and in-session measures used for comorbid conditions.

Condition	Baseline, 6-month, and 12-month measures	Additional in-session measures
Depression	Patient Health Questionnaire-9 (PHQ-9) (37); a score of ≥10 was used to identify moderate to severe depression	PHQ-9 was used to track depression symptoms. Specialty referral was recommended for scores >20.
Anxiety	Brief Symptom Inventory (BSI) Anxiety subscale (38–41); a T-score of ≥63 was used to identify clinically significant anxiety	BSI Anxiety subscale was used to track anxiety and PTSD symptoms. Specialty referral was recommended for scores >70. Items from the Anxiety and Depression Detector (42) were used to identify symptoms of panic, generalized anxiety, social anxiety, and PTSD.
Post-traumatic Stress Disorder	PTSD Checklist-Military version (PCL-M) (43); a global score of ≥50 was used to identify probable PTSD	
Insomnia	Pittsburgh Sleep Quality Inventory (PSQI) (44–46); a score of ≥9 was used to identify moderate sleep disturbance.	Insomnia Severity Index (ISI) (47) was used to track insomnia symptoms. Specialty or PCP referral was recommended for scores >21.
Headache	Rivermead Postconcussion Symptoms Questionnaire (RPQ) (48); a score of ≥3 on the headache item indicates moderate to severe headaches.	Headache Impact Test (HIT-6) (49) was used to track headache symptoms. Specialty referral was recommended for scores >60.

### Interventions

Both treatment arms included 178 randomly assigned participants. All participants received a study binder that included educational brochures on problems commonly experienced by Service Members returning from deployment (e.g., cognitive deficits, finances, sleep disturbance). Participants in the EO condition received a second copy of the brochures by mail, one every two weeks.

The PST intervention consisted of 12 scheduled biweekly calls placed by Masters-trained counselors, called Concussion Support Specialists (CSS), who were trained and supervised by a psychiatrist, licensed psychologist and physicians throughout the study. During these calls, service members learned and practiced a manualized 6-step strategy for selecting, characterizing, and solving problems affecting their daily lives (16). In addition, clinical status was monitored during each call for elevated symptoms of each of the five comorbidities. If participants reported clinically significant depression, anxiety, PTSD, sleep disturbance, or headache, the CSS suggested augmenting the PST intervention with brief, comorbidity-specific interventions, or modules, each designed to span 2–4 sessions. These modules, which were also manualized, included additional assessment, education, and evidence-based therapeutic strategies for each comorbidity. We used principles of behavioral activation (BA) (18–22) for the depression and anxiety/PTSD modules, with content adapted for the specific disorder; psychoeducation (including stimulus control and sleep hygiene) and components of cognitive behavioral therapy for insomnia (CBT-I) (23–25); and psychoeducation and mindfulness-based strategies (26–29) for the headache module. Problem-specific educational packets and worksheets were provided and symptoms were monitored using the validated instruments shown in Table 1. After completing a module, the CSS continued to monitor and support the problem during the remainder of the intervention and, when indicated, recommended referral for further treatment.

### Data analysis

We examined the effect of the interventions on the severity of comorbidities at 6 and 12 months using generalized estimating equations to estimate the parameters of a generalized linear model (30). We analyzed the effect of the intervention on the prevalence of dichotomous comorbidity outcomes by examining the number and percent of those in the PST and EO groups with each of the comorbidities at baseline, 6 months, and 12 months. Values of missing items on individual instruments were imputed by prorating if at least half of the items were completed for that instrument. We also identified the proportion of those who were responders, defined as those who screened positive for a comorbidity at baseline but did not meet case criteria for that comorbidity at the 6- or 12-month outcome assessment. Differences in prevalence between the PST and EO groups were assessed using mixed-effects logistic regression, which safeguards against any potential bias due to unobserved outcomes under the assumption that they are missing at random (MAR). Differences in response were assessed using exact logistic regression. Site, military status (active duty vs. National Guard/ reserve) and the baseline Brief Symptom Inventory (31). Global Severity Index T-score were used as covariates in the analyses. We considered the analysis of each comorbidity to provide crucial information independent of the other analyses. Because the risk of Type II error was as important as the risk of Type I error in this instance, we set alpha = 0.05 for each statistical comparison.

Using the dichotomous comorbidity outcomes, we created a variable indicating the number of comorbidities that each participant had at baseline, 6 months, and 12 months. This variable was classified as missing for a given time point if the participant did not complete all 5 measures. We used SPSS (version 19) and SAS (version 9.3) for all analyses.

## Results

### Participant characteristics

Characteristics of the sample recruited into the parent trial and the flow of participants from screening to follow-up are reported elsewhere (17). In brief, trial participants were 29 years old on average, 93% male, 77% white, and had a mean education level of 13.4 years. The average participant had undergone 2–3 deployments, and the majority (73%) had sustained 3 or more mTBIs, mostly involving blast injury. As previously reported, there were no significant differences between the groups in their baseline demographic, injury, or clinical characteristics (17). At the 6 month assessment, 78% of the PST participants and 93% of the EO participants provided outcomes data; at 12 months, the follow-up rates were 80 and 88%, respectively. The mean number of treatment sessions completed by the PST participants was 6.6 (SD 4.6, range 0–12, median 7); 119 (67%) completed 4 or more sessions.

### Comorbid conditions

Table 2 shows the numbers and proportions of participants who screened positive for each comorbid condition at baseline; the groups were equivalent on these measures. Nearly half of participants endorsed three or more comorbidities. Sleep disturbance was the most common (80%), followed by headache (57%) and depression (50%). Among the 62 participants with one comorbidity, sleep disturbance was the most common (*n* = 42); among the 86 participants with two comorbidities, sleep disturbance and headache was the most common combination (*n* = 55); among the 66 participants with three comorbidities, the combination of sleep disturbance, headache, and depression was the most common (*n* = 33); among the 48 participants with four comorbidities, the combination of sleep disturbance, depression, anxiety, and PTSD was the most common (*n* = 24).

**Table 2:** Comorbidities at baseline by treatment group.

Variable	Randomized to EO (*n* = 178)	Randomized to PST (*n* = 178)	*p*-value^a^
Comorbidity type^b^ – *n* (%)			
Depression – PHQ-9 ≥ 10	86 (48)	91 (51)	0.37
Anxiety – BSI anxiety T-score ≥63	61 (34)	74 (42)	0.19
PTSD – PCL-M global ≥50	50 (28)	50 (28)	1.00
Sleep disturbance – PSQI global ≥9	146 (82)	138 (78)	0.43
Headache – RPQ headache item ≥3	103 (58)	100 (56)	0.83
Total comorbidities – *n* (%)			
Mean (SD)	2.5 (1.5)	2.6 (1.6)	0.76
0	16 (9)	22 (12)	0.52
1	35 (20)	27 (15)	
2	43 (24)	43 (24)	
3	34 (19)	32 (18)	
4	27 (15)	21 (12)	
5	23 (13)	32 (18)	

EO, education only; PHQ-9, Patient Health Questionnaire-9 depression scale; PCL-M, PTSD Checklist-Military version; PSQI, Pittsburgh Sleep Quality Inventory; PST, problem solving treatment; PTSD, post-traumatic stress disorder; SD, standard deviation; RPQ, Rivermead Post-concussion Symptom Questionnaire.

a Significance by Fisher Exact or Mann–Whitney U test.

b 1 missing PHQ-9, 2 missing PSQI, 1 missing RPQ headache.

Results of the analysis of comorbidity outcome measures at 6 and 12 months are summarized in Table 3. Depression, anxiety, PTSD and sleep were significantly improved in the PST group at 6 months, compared to the EO group, while headache was not. The mean number of comorbidities was also significantly lower in the PST group compared to the EO group at 6 months. Among the PCL-M subscales, re-experiencing symptoms were significantly improved in the PST group compared to the EO group (*p* < 0.05). There were no statistically significant differences between the PST and EO groups for any of the comorbidity outcomes or the total number of comorbidities at 12 months.

**Table 3:** Comorbidity outcomes by treatment groups at 6 and 12 months^a^.

Comorbidity	Month	*N*	Mean (SD)		Adj. difference^b^	95% CI	*p*-value
			EO	PST			
Depression PHQ-9	0	355	10.0 (5.3)	10.1 (5.4)	0.04	(−0.8, 0.9)	0.922
	6	284	9.2 (5.7)	7.6 (6.2)	**−1.4**	**(−2.6, −0.1)**	**0.030**
	12	266	8.4 (5.8)	8.2 (6.4)	−0.2	(−1.5, 1.2)	0.822
Anxiety BSI anxiety	0	356	5.9 (5.4)	6.6 (5.5)	0.6	(−0.2, 1.4)	0.143
	6	304	6.4 (5.8)	5.5 (5.8)	**−1.6**	**(−2.7, −0.4)**	**0.007**
	12	298	6.2 (5.6)	6.5 (6.1)	−0.5	(−1.8, 0.8)	0.489
PTSD PCL-M global score	0	356	41.6 (14.2)	41.3 (14.3)	−0.5	(−2.6, 1.7)	0.688
	6	292	42.0 (16.1)	38.7 (17.2)	**−3.0**	**(−5.7, −0.2)**	**0.036**
	12	271	40.7 (15.9)	39.5 (17.2)	−1.1	(−4.3, 2.1)	0.485
Sleep disturbance PSQI global score	0	352	12.6 (4.1)	12.5 (4.5)	−0.2	(−1.0, 0.7)	0.710
	6	276	11.8 (4.7)	10.1 (5.0)	**−1.3**	**(−2.3, −0.3)**	**0.015**
	12	255	10.8 (4.9)	10.7 (5.5)	−0.01	(−1.1, 1.1)	0.989
Headache/pain RPQ headache item	0	355	2.6 (1.2)	2.55 (1.2)	−0.01	(−0.3, 0.2)	0.954
	6	304	2.2 (1.3)	2.18 (1.4)	−0.02	(−0.3, 0.2)	0.889
	12	299	2.2 (1.2)	1.97 (1.3)	−0.2	(−0.5, 0.1)	0.143
Number of comorbidities (0–5)	0	355	2.5 (1.5)	2.56 (1.6)	0.04	(−0.2, 0.3)	0.722
	6	292	2.4 (1.7)	2.05 (1.8)	**−0.4**	**(−0.7, −0.03)**	**0.035**
	12	271	2.1 (1.7)	2.18 (1.8)	0.00	(−0.4, 0.4)	0.994

BSI-Anxiety, Brief Symptom Inventory-Anxiety scale; EO, education only; PHQ-9, Patient Health Questionnaire-9 depression scale; PSQI, Pittsburgh Sleep Quality Inventory; PST, problem solving treatment; RPQ, Rivermead Postconcussion Symptom Questionnaire.

a Baseline *p*-values are from a standard regression model, while 6-month and 12-month P-values reflect time-by-treatment interactions from a generalized linear model with parameters estimated using generalized estimating equations (GEE). All models adjusted for site, military status, and baseline BSI global score. *N* represents the total number of participants who completed the measure at each assessment.

b For month 0, the difference reported is between EO and PST groups adjusted for site, military status, and baseline BSI global score. For months 6 and 12, the difference reported is the difference from baseline to 6- and 12-months between EO and PST groups, adjusted for site, military status, and baseline BSI global score.Bold values indicate *p*<0.05.

Table 4 shows the prevalence and response rates for each comorbidity at 6 and 12 months. Depression and sleep disturbance were both significantly less prevalent at 6 months in the PST group than the EO group. Similarly, among those with the respective comorbidity at baseline, more PST participants than EO participants were responders (i.e., no longer met case criteria) for depression (48% vs. 27%, *p* = 0.005) and sleep disturbance (31% vs. 16%, *p* = 0.013) at 6 months. At 6 months, the PST group also demonstrated significantly higher remission rates than the EO group for depression (PHQ-9 < 5; 22% vs. 8%, *p* = 0.026) and sleep disturbance (PSQI < 6; 15% vs. 5%, *p* = 0.017). There were no significant differences in prevalence, response, or remission rates at 12 months.

**Table 4:** Prevalence of comorbidities and proportion of responders at 6 and 12 months.

Comorbidity	Month	N	EO	PST	Adj. OR	95% CI	*p*-value
Prevalence^a^							
Depression	6	284	74 (47%)	42 (33%)	**0.46**	**(0.25, 0.87)**	**0.016**
	12	266	51 (37%)	47 (37%)	0.85	(0.45, 1.63)	0.633
Anxiety	6	304	62 (37%)	51 (37%)	0.67	(0.34, 1.29)	0.228
	12	298	55 (35%)	58 (41%)	0.92	(0.45, 1.86)	0.809
PTSD	6	292	55 (34%)	38 (29%)	0.79	(0.42, 1.48)	0.460
	12	271	39 (28%)	43 (33%)	1.44	(0.74, 2.82)	0.285
Sleep Disturbance	6	276	119 (77%)	71 (58%)	**0.53**	**(0.29, 0.97)**	**0.041**
	12	255	95 (70%)	84 (70%)	1.14	(0.64, 2.02)	0.648
Headache	6	304	78 (47%)	64 (46%)	1.06	(0.64, 1.75)	0.813
	12	299	65 (41%)	56 (39%)	0.96	(0.55, 1.70)	0.897
Response^b^							
Depression	6	141	21 (27%)	31 (48%)	**3.01**	**(1.39, 6.52)**	**0.005**
	12	127	24 (38%)	29 (46%)	1.42	(0.68, 2.95)	0.461
Anxiety	6	107	17 (31%)	18 (34%)	1.06	(0.46, 2.43)	1.000
	12	106	23 (46%)	17 (30%)	0.49	(0.22, 1.12)	0.106
PTSD	6	80	12 (27%)	11 (31%)	1.20	(0.45, 3.22)	0.806
	12	70	14 (38%)	9 (27%)	0.67	(0.23, 1.90)	0.606
Sleep disturbance	6	219	20 (16%)	29 (31%)	**2.33**	**(1.21, 4.49)**	**0.013**
	12	205	20 (18%)	19 (20%)	1.13	(0.56, 2.27)	0.859
Headache	6	171	32 (34%)	29 (38%)	1.22	(0.65, 2.30)	0.631
	12	168	45 (50%)	39 (50%)	1.00	(0.53, 1.86)	1.000

EO, education only; PST, problem solving treatment; PTSD, post-traumatic stress disorder.

a Presence of comorbidity is defined as: Depression: PHQ-9 ≥ 10; Anxiety: BSI-Anxiety T-score ≥ 63; PTSD: PCL-M Global ≥ 50; Sleep Disturbance: PSQI Global ≥ 9; Headache: RPQ Headache Item ≥ 3. Prevalence estimates based on mixed-effects regression models that adjust for site, military status, and baseline BSI global score. *N* represents the total number of participants who completed the measure at each assessment.

b Response is defined as no longer meeting criteria for a comorbidity at 6- or 12-month follow-up among those who met case criteria for the comorbidity at baseline. Estimates are based on exact logistic regression models that adjust for site, military status, and baseline BSI global score. *N* represents the total number of participants who had the comorbidity at baseline and completed the measure at each assessment.Bold values indicate *p*<0.05.

Examining the sleep disturbance and headache findings in greater detail, 43 participants chose to participate in the “insomnia module” for sleep disturbance (mean 3.6 ± 1.6 sessions) and only 3 participated in the “headache module” (mean 4.3 ± 0.6 sessions). Among participants who had significant sleep disturbance on the PSQI at baseline, those who received the insomnia module had higher mean baseline scores than those who did not (13.9 ± 3.9 vs. 12.0 ± 4.6, *p* < 0.001), with scores of 11.0 ± 5.2 at 6 months and 12.1 ± 6.4 at 12 months. The in-session ISI score significantly improved among participants in the insomnia module, with the mean dropping from 17.0 ± 5.0 to 11.3 ± 6.5, (*p* < 0.001). There was no significant improvement in mean HIT-6 scores among the 3 headache module participants (64 ± 10 to 63 ± 17, *p* = 0.84). Similar data are not available for the depression and anxiety/PTSD modules because components of these modules were used by the counselors and integrated throughout the intervention in response to evidence of depression or anxiety/PTSD on routine in-session screening for overall distress, depression and anxiety.

## Discussion

In a previous report on the efficacy of a telehealth intervention for service members with mTBI, we focused on group means to show that a problem-solving treatment (PST) was superior to education alone (EO) for reducing psychological distress, but not post-concussive symptoms (17). This secondary analysis, focused on the comorbidities that often accompany mTBI, provides further information on the impact of using a person-centered modular approach to supplement the general problem-solving approach in the active treatment arm. We found that this approach significantly improved both the number and the symptom burden of multiple common comorbidities and overall symptom burden. Specifically, symptoms of depression, anxiety, PTSD, and sleep disturbance all improved after the end of the 6-month treatment period, and the proportion of service members with clinically significant symptoms of depression and sleep disturbance also significantly decreased from baseline to 6 months in the group receiving this modular treatment. Unfortunately, superiority of the PST group was not maintained to the 12-month assessment as comorbidities and symptoms were equivalent to the EO group at this timepoint. These results are similar to the follow-up findings in the parent trial (17). Additionally, headache pain improved equally in both groups.

Our study sample exhibited a high baseline level of symptomatology in the five comorbidities of interest, despite not requiring any symptoms for enrollment. The improvement in both the number of comorbidities and the proportion of service members with clinically significant symptoms may translate into improved readiness to return to duty and reduced burden on healthcare systems. The fact that multiple, potentially debilitating, inter-related symptoms showed improvement suggests that this brief, flexible treatment model could be adapted to the complex array of problems experienced by Veterans of overseas conflicts, with the possible exception of headache. The reasons why headache did not show a greater treatment response in the PST group need to be further explored; meanwhile, inclusion of a more traditional medical management approach for headache may be appropriate.

Similar to prior findings (17), these results support the use of the telephone to extend the clinical reach of behavioral interventions to service members; two-thirds of our participants completed at least four sessions, which has been cited as a “minimally effective dose” of psychotherapy in some studies (32, 33). Even for comorbidities such as these, which are quite disruptive to daily functioning and characterized as difficult to treat (34), telehealth interventions hold promise for reducing barriers to evidence-based care by overcoming stigma, lack of access, transportation difficulties, and avoidance behaviors. A qualitative study conducted at the close of the parent trial confirmed that very few participants would have preferred face-to-face treatment (35).

For the comorbidities that showed improvement with modular treatment at 6 months (depression, anxiety, PTSD, and sleep disturbance), the reason for the lack of group differences at 12 months appears to be two-fold. The PST group showed some relapse in symptom severity between 6 and 12 months, while the EO group showed symptom improvement during the same interval. More research is needed to elucidate the reasons for the symptom relapse after completion of the intervention and to explore strategies for maintaining the initial gains, such as booster sessions, or “apps” for reminding participants of personally effective strategies.

Several limitations of this study should be noted when interpreting results. First, most participants were male and served exclusively in the Army, with few National Guard and reserve members represented. Gender differences in treatment response would be valuable to explore in future research. Second, our loss to follow-up rate was higher in the PST group. Several reasons for this may be possible, including dropping out due to fatigue from the number of calls or feeling that their outcomes were already being assessed as part of the PST intervention. Third, we lack detailed data on referrals made as a result of participation in the modules, which could have affected treatment response; however, an analysis examining the impact of the PST intervention on healthcare utilization did show increased use of hospital services (36). Finally, we enrolled participants on the basis of their having sustained mTBI during deployment, rather than on the basis of symptom severity. Results may have differed had we attempted to recruit participants with a minimum level of symptomatology at baseline.

In conclusion, telephone-based PST, supplemented with brief, condition-specific modules to reduce the cumulative burden of comorbidities such as depression, anxiety, PTSD and sleep disturbance is a promising approach for mTBI in military populations. Future studies should extend this approach to more diverse Veteran and civilian populations with mTBI. It would also be helpful to develop and test brief modules for other common comorbidities, such as cognitive problems and anger/ irritability.

## Data Availability

The raw data supporting the conclusions of this article will be made available by the authors, without undue reservation.

## Group member of the CONTACT investigators

The CONTACT study team members: Jef St. De Lore, Sara E. Fey-Hinckley, Jocelyn L. Savage, and Michael Warren, University of Washington, Seattle, WA; Gregory Gahm, Elissa Thomas, Karina Boykin, and Derek Smolensky, National Center for Telehealth and Technology, T2, Tacoma, WA; Leila Forbes and Danielle Benton, Defense and Veterans Brain Injury Center, now the TBI Center of Excellence; and the Geneva Foundation, Tacoma, WA, in particular Kasey Zink.
